# Commentary: New Viscoelastic Hydrogel Hymovis MORE Single Intra-Articular Injection for the Treatment of Knee Osteoarthritis in Sportsmen: Safety and Efficacy Study Results

**DOI:** 10.3389/fphar.2021.785074

**Published:** 2021-12-22

**Authors:** Thierry Conrozier, Thomas Lohse

**Affiliations:** Nord Franche-Comté Hospital, Belfort, France

**Keywords:** viscosupplementation, knee, osteoarthritis, hyaluronic acid, intra-articular injection

**A Commentary on**

**New Viscoelastic Hydrogel Hymovis MORE Single Intra-Articular Injection for the Treatment of Knee Osteoarthritis in Sportsmen: Safety and Efficacy Study Results**

*by Bernetti, A., Agostini, F., Alviti, F., Giordan, N., Martella, F., and Santilli, V. (2021). Front Pharmacol. 12:673988. doi:* *10.3389/fphar.2021.673988*

We read with interest the recent article of Bernetti et al. (2021) showing that a single Hymovis MORE intra-articular (IA) injection provides a rapid and long lasting response in sportsmen suffering from knee osteoarthritis (OA). Such a result could appear surprising in view of the low dose of hyaluronic acid (HA) injected (32 mg). In fact, the most frequently used doses are in the range of 60 mg, whether the treatment is administered as repeated injections or as a single injection. Viscosupplementation by IA injection of a HA solution is a validated symptomatic treatment for mild to moderate knee OA. However, although the treatment is recommended, at least under certain conditions, by the majority of learned societies (Zhang et al., 2005; Abdulla et al., 2013; Bannuru et al., 2019; Bruyère et al., 2019; Sellam et al., 2020) there is curiously no consensus concerning the ideal dosage of HA to be injected. If the total dose of HA administered is usually 60 mg with 3-injection protocols (3 times 20 mg), the dosing regimen can vary according to a ratio of 1–5 with HA in mono-injections, ranging from 32 mg (Hymovis^©^, Fidia Laboratory, Italy) for the least concentrated to 120 mg for the highest dosage product (Solo120^©^, Ettsons Laboratory, France). One can thus wonder about the relevance of such differences in dosage, knowing that the HA molecule remains the same from one formulation to another, varying only by its molecular weight and its three-dimensional configuration, reticulated or linear.

Although several meta-analyses emphasize the interest of a higher molecular weight (Altman et al., 2016; Webner et al., 2021; Wu et al., 2021), none of them mention differences in efficacy related to the total dose injected. This seems logical when one considers that a healthy knee contains on average only 4–10 mg of HA (Balazs and Denlinger, 1993) and that experimental animal models have identified a regulatory mechanism that involves an increase in joint clearance of HA based on the dose injected (Balazs and Denlinger, 1993). More simply, the more HA is injected, the more its elimination is accelerated in order to restore a physiological HA concentration level when it is artificially increased. For Balazs et al., the precursors of the viscosupplementation concept, the effectiveness of a viscosupplement depends on its rheological properties and its residence time in the joint tissues (Balazs and Denlinger, 1993). The latter is brief (2–3 days) for HA with a linear structure (Lindenhayn et al., 1997) and up to 10 times longer for cross-linked HA (Lindqvist et al., 2002; Larsen et al., 2012). No work has yet demonstrated that increasing the volume injected or the concentration of HA can prolong the intra-articular residence time of HA, or improve the symptomatic efficacy. Only one *in vitro* study (Boissier et al., 2020) has shown a superior chondroprotective effect of a higher dose of HA (SINOVIAL^®^, Laboratoires Genévrier, France) compared with the same HA in twice the concentration. However, it is difficult to extrapolate these results to the clinic, since this study does not take into account the rate of elimination of the viscosupplement.

To support our hypothesis that the amount of HA does not significantly influence clinical outcome, we performed a post hoc analysis of 2 prospective studies, performed under strictly similar conditions in patients treated with HA monoinjection for symptomatic gonarthrosis. In Study 1 (Conrozier et al., 2018), the patients received a single dose of 2.2 ml of HANOX-M-XL (Happycross^®^, Laboratoire LABRHA, France), a viscosupplement consisting of 16 mg/ml of cross-linked HA, coupled with 35 mg/ml of mannitol. In Study 2 (Conrozier et al., 2016), comparable patients received 2 doses (4.4 ml) of the same viscosupplement during the same session. Thus, the former received 35.2 mg HA and the latter 70.4 mg. The populations on the day of injection (D0) and the clinical results at 6 months (M6) were compared. Efficacy criteria were change in WOMAC index (A pain, C function), patient global assessment of pain (PGA), and patient rating of treatment effectiveness (poor, fair, good, very good). Adverse events attributable to treatment were compared. A total of 93 patients (53 in study 1 and 40 in study 2) were analyzed. At D0 the populations of the 2 studies were strictly comparable in terms of demographics and clinical characteristics. At M6 there was a significant improvement in pain (*p* < 0.0001), function (*p* < 0.0001) and PGE (*p* < 0.001) in both studies, with no difference between them (Table 1). Efficacy was rated as very good or good in 77.3 and 76.9%, respectively. Local adverse events were reported in 5.7 and 5% of patients.

**TABLE 1:** Characteristics of patients (N = 93) treated with a single-dose (N = 53) or a double-dose (N = 40) of HANOX-M-XL (HAPPYCROSS^®^) for painful knee osteoarthritis.

Variables (DS)	Single dose *N* = 53	Double dose *N* = 40	*p*-values
Age (years)	62.6 (12.3)	60.7 (13.9)	0.58
BMI (kg/m^2^)	27.5 (5.2)	28.6 (5.0)	0.89
Duration (months)	54.0 (36.5)	44.5 (18.0)	0.18
WOMAC A at D0 (0–10)	4.5 (1.2)	4.3 (1.9)	0.69
WOMAC C at D0 (0–10)	3.9 (1.2))	3.4 (1.9)	0.63
Global assessment D 0 (0–10)	6.0 (1.1)	5.5 (2.0)	0.54
WOMAC A at D180 (0–10)	1.83 (1.05)	2.5 (2.5)	0.19
WOMAC C at D180 (0–10)	1.91 (1.2)	2.1 (2.3)	0.75
Global assessment D180 (0–10)	3.1 (1.5)	3.0 (2.1)	0.89

Although this was not a head-to-head study and the number of patients studied was small, this analysis strongly suggests that increasing the amount of HA injected does not provide any clinical benefit in terms of pain or function, nor does it increase the risk of adverse effects. It therefore seems pointless to increase the doses, volume or concentration in case of failure of a previous viscosupplementation treatment. Further studies are needed to determine the “optimal” dose of HA to be injected, which may depend on the molecular weight and likely on the IA residence time of the device, but which might be much lower than those currently used.
